# Macronutrient Distribution and Dietary Sources in the Spanish Population: Findings from the ANIBES Study

**DOI:** 10.3390/nu8030177

**Published:** 2016-03-22

**Authors:** Emma Ruiz, José Manuel Ávila, Teresa Valero, Susana del Pozo, Paula Rodriguez, Javier Aranceta-Bartrina, Ángel Gil, Marcela González-Gross, Rosa M. Ortega, Lluis Serra-Majem, Gregorio Varela-Moreiras

**Affiliations:** 1 Spanish Nutrition Foundation (FEN), C/General Álvarez de Castro 20, 1 pta, Madrid 28010, Spain; eruiz@fen.org.es (E.R.); jmavila@fen.org.es (J.M.Á.); tvalero@fen.org.es (T.V.); susanadelpozo@fen.org.es (S.P.); prodriguez@fen.org.es (P.R.); 2 Department of Preventive Medicine and Public Health, University of Navarra, C/Irunlarrea 1, Pamplona 31008, Spain; jaranceta@unav.es or javieraranceta@hotmail.com; 3 Department of Biochemistry and Molecular Biology II, Institute of Nutrition and Food Sciences, University of Granada. Campus de la Salud, Avda. del Conocimiento, Armilla, Granada 18100, Spain; agil@ugr.es; 4 ImFINE Research Group, Department of Health and Human Performance, Technical University of Madrid, C/Martín Fierro 7, Madrid 28040, Spain; marcela.gonzalez.gross@upm.es; 5 Department of Nutrition, Faculty of Pharmacy, Complutense University of Madrid, Plaza Ramón y Cajal s/n, Madrid 28040, Spain; rortega@ucm.es; 6 Research Institute of Biomedical and Health Sciences, Universidad de Las Palmas de Gran Canaria, Facultad de Ciencias de la Salud, C/Doctor Pasteur s/n Trasera del Hospital, Las Palmas de Gran Canaria, Las Palmas 35016, Spain; lluis.serra@ulpgc.es; 7 Department of Pharmaceutical and Health Sciences, Faculty of Pharmacy, CEU San Pablo University, Urb. Montepríncipe, Crta. Boadilla Km 53, Boadilla del Monte, Madrid 28668, Spain

**Keywords:** macronutrient intake, dietary protein, dietary fat, carbohydrate intake, dietary fat quality sources, ANIBES study

## Abstract

Our aim was to analyze dietary macronutrient intake and its main sources according to sex and age. Results were derived from the ANIBES (“Anthropometry, Intake and Energy Balance in Spain”) cross-sectional study using a nationally-representative sample of the Spanish population (9–75 years old). Mean dietary protein intake was 74.5 ± 22.4 g/day, with meat and meat products as the main sources (33.0%). Mean carbohydrate intake was 185.4 ± 60.9 g/day and was higher in children and adolescents; grains (49%), mainly bread, were the main contributor. Milk and dairy products (23%) ranked first for sugar intake. Mean lipid intake was 78.1 ± 26.1 g/day and was higher in younger age groups; contributions were mainly from oils and fats (32.5%; olive oil 25.6%) and meat and meat products (22.0%). Lipid profiles showed relatively high monounsaturated fatty acid intake, of which olive oil contributed 38.8%. Saturated fatty acids were mainly (>70%) combined from meat and meat products, milk and dairy products and oils and fats. Polyunsaturated fatty acids were mainly from oils and fats (31.5%). The macronutrient intake and distribution in the Spanish population is far from population reference intakes and nutritional goals, especially for children and adolescents.

## 1. Introduction

Obesity and other nutrition-related non-communicable diseases represent increasing major health problems in Mediterranean countries, such as Spain [1]. Being overweight and obesity affect more than 50% of adults and nearly 30% of children in Spain [2].

Undoubtedly, deep social and economic changes occurred in this country in the last few decades, which also experienced a transition in dietary patterns and life styles [1,2,3]. Some have had a potentially positive impact, such as increasing the variety of foods, access (in fact, a potential overabundance of energy and nutrients) and food security in the diet. However, globally, these changes are contradictory with adequate food selection and adherence for a healthy Mediterranean diet [4].

The health-promoting quality of the overall diet is usually associated with energy and nutrient intake. Populations are encouraged to meet their energy and nutrients needs primarily through foods [5]. National dietary surveillance, while having inherent limitations (misreporting, accurate updating of food composition tables at the national level, *etc*.), provides a way to examine eating patterns and their impact on calorie and nutrient intakes across different populations [6,7,8].

The use of new available methodologies (e.g., real-time recording of eating and drinking events) has been urgently claimed to avoid these difficulties [9,10,11]. The latter was firmly stated in the consensus document and conclusions “Obesity and sedentarism in the 21st century: What can be done and what must be done?” [4] and even more recently in the “Methodology of dietary surveys, studies on nutrition, physical activity and other lifestyles” special review supplement [12]**.**

Different dietary surveys have been previously conducted in Spain [13,14,15,16]. However, no one has approached up to date energy and macronutrients intake using new, more accurate technologies. As a consequence, the ANIBES (“Anthropometry, Intake and Energy Balance in Spain”) study was recently completed. We have previously reported the ANIBES design and methodology [17,18] and energy intake and its dietary sources [19].

This study focuses on macronutrient intake in the Spanish diet, to better characterize also the macronutrient excess or inadequacy, as well as to analyze food and beverage sources that currently contribute to the dietary intake of carbohydrates, lipids and proteins. The latter aim is of particular interest, to provide more detailed and accurate information on how the different food and beverage groups and subgroups represent the current market in Spain. Moreover, targeting current food and beverage selections among the Spanish population is key to the design of the dietary guidelines and public health strategies to improve the diet quality in the future.

## 2. Materials and Methods

The complete design, protocol and methodology of the ANIBES study have been described in detail elsewhere [17,18].

### 2.1. Sample

The initial potential sample consisted of 2634 individuals, and the final sample comprised 2009 individuals (1013 men, 50.4%; 996 women, 49.6%). In addition, for the youngest age groups (9–12, 13–17 and 18–24 years), a boost sample was included to have at least *n* = 200 per age group (error ±6.9%). Therefore, the random sample plus booster comprised 2285 participants. Sample quotas according to the following variables were: age groups (9–12, 13–17, 18–64 and 65–75 years); sex (men/women); geographical distribution (northeast, Levant, south, central, northwest, Barcelona metropolitan area, Madrid metropolitan area and Balearic and Canary Islands); and locality size: 2000–30,000 inhabitants (rural), 30,000–200,000 inhabitants (semi-urban) and over 200,000 inhabitants (urban).

The final protocol was approved by the Ethical Committee for Clinical Research of the Region of Madrid in Spain.

### 2.2. Food and Beverage Records

Study participants were provided with a tablet device (Samsung Galaxy Tab 27.0) to record by taking photos of all food and drinks consumed during 3 days, both at home and outside. Photos had to be taken before beginning to eat and drink and again after finishing, so as to record the actual intake. The ANIBES software was developed to receive information from the field tablets every 2 seconds, and the database was updated every 30 min. Food, beverages and nutrient intakes were calculated from food consumption records using software (VD-FEN 2.1), which is based mainly on Spanish food composition tables [20]. Macronutrient reference intakes and distribution objectives for the Spanish population were used to analyze the overall quality of the diet [21].

### 2.3. Statistical Analysis

The intake data were grouped into 16 food groups, 38 subgroups and 754 ingredients for in-depth analysis. Every comparison between groups has been performed by a Student’s *t*-test for independent samples with a 95% confidence interval. In addition, the Kolmogorov–Smirnoff normality test was used to test the normality of the distribution: random sample (2009 participants) and random + booster sample (2285). The random sample is used to show total sample data and to compare between sexes. To compare age groups and sex in age groups, a booster sample was included in order to expand those age groups less represented in the random sample.

## 3. Results

### 3.1. Macronutrient Intake and Distribution

Daily intake levels of macronutrients, alcohol, water and their distributions are shown in Table 1. The mean protein intake was 74.5 ± 22.4 g/day, ranging from 28.2 to 352.5 g/day. Differences were observed between men and women and also according to the age group (the oldest showed the lowest intake), as shown in Table 2.

The mean total carbohydrate intake was 185.4 ± 60.9 g/day (37.8 g/day min; 450.3 g/day max) (Table 1), with differences seen between men and women, as also shown in Table 2. Higher total carbohydrate consumption was observed in younger age groups as compared to adults and older adults. Total sugar intake was also quantified: higher in children and adolescents and markedly lower in adults and older adults. Fiber intake and distribution are also shown in Table 1, with 12.7 ± 5.6 g/day (2.2 g/day min; 45.1 g/day max), and differences were also found between men and women; values were also much higher in older adults than in the youngest populations.

Mean lipid intake and distribution are shown in Table 1, with 78.1 ± 26.1 g/day, and ranging from 21.0 g/day to 201.5 g/day. Results for the different age groups are shown in Table 2. Values were much higher among younger age groups than older adults. Sex differences were also observed, being higher in men in all age groups. A decreasing trend in intake with advancing age is observed.

### 3.2. Contribution of Food and Beverage Groups/Subgroups to Total Macronutrients

**Figure 1:**
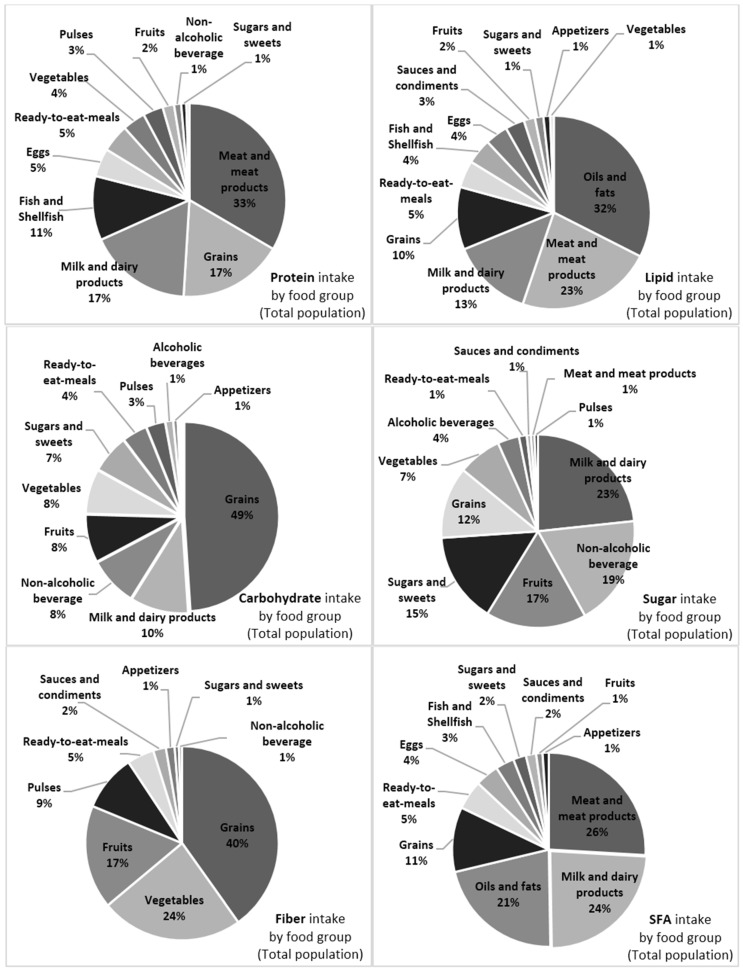

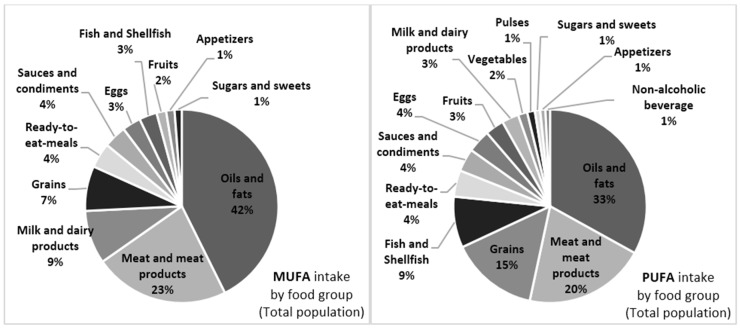
Nutrient intake (%) by food group in the Spanish ANIBES population aged 9–75 years. Food groups that contribute <1% of the diet to the nutrient are not shown. SFA: saturated fatty acids; MUFA: monounsaturated fatty acids; PUFA: polyunsaturated fatty acids.

Contributions (%) of the various food and beverage categories to daily macronutrient intake is shown in Figure 1. More detailed information according to different age groups (9–12, 13–17, 18–64 and 65–75 years) is provided in Table 3, Table 4, Table 5, Table 6 and Table 7.

Meat and meat products were the main sources of protein for the whole population (33.1%), although these contributed much more among younger groups and less so among older adults. Grains and milk and dairy products ranked second and third, with these groups together contributing over two-thirds (68%) of the total protein intake (Figure 1). Other protein-rich foods were fish and shellfish, much higher in older adults. Interestingly, vegetables and pulses contributed only 7% to total daily protein intake and were especially low in the youngest age groups.

Carbohydrate intake in the Spanish diet was mainly from grains, with bread as the main contributor (Figure 1). Within this group, baked goods and pastries ranked next, and this category was higher for children than for adults and older adults. Grains were followed to a much lesser extent by milk and dairy products, nonalcoholic beverages, fruits, vegetables, sugars and sweets and with much lower contributions from ready-to-eat meals, pulses, alcoholic beverages and appetizers (together accounting for 10% of the total). Older adults had a better variety of carbohydrate sources compared to children and adolescents, mainly owing to a higher intake of fruits, vegetables and pulses. Contrarily, the youngest groups showed a much higher contribution from sugars and sweets and nonalcoholic beverages. Considering sugar intake by food group, milk and dairy products were the main contributors, followed by fruits, sugars and sweets, grains and much lower contributions from vegetables and alcoholic beverages; minor contributors were ready-to-eat meals, sauces and condiments, meat and meat products and pulses, with roughly 1% each. Dietary fiber in the current Spanish diet was found in descending order to be mainly from grains, vegetables, fruits and pulses.

Contributions of food groups and subgroups to the total lipid intake are also shown in Figure 1. Oils and fats represented the main source, especially from olive oil (see Table 3). Meats and meat products ranked second, with contributions mainly from meat, but almost equally as much from sausages and other meat products. The next food group contributor was milk and milk products, with cheeses as the main subgroup. Grains, mainly baked goods and pastries, were the fourth contributor to lipid intake, whereas the remaining groups only contributed from 1% to 5%. Differences were found according to age group: olive oil intake was much higher among older adults (33.2%) than among children or adolescents (roughly 18%); fish and shellfish intake also increased with increasing age. Ready-to-eat meal consumption was, however, higher among the youngest groups. Finally, intake of sausages and other meat products was higher in younger groups, whereas children and adolescents accounted for the lowest consumption of meats.

An even more important issue was understanding how the different food and beverage groups and subgroups were contributing to the quality of dietary fat, namely, SFA, MUFA and PUFA, including *n*-6 and *n*-3 fatty acids (Figure 1).

SFA were obtained primarily (>70%) and almost equally from meat and meat products, milk and dairy products and oils and fats. In younger age groups, the greatest contribution came from the sausage and meat derivatives subgroup, followed by bakery and pastry. In adults and older adults, olive oil and meat ranked as the primary contributors.

As for MUFA intake, oils and fats were the greatest contributor, of which olive oil accounted for 36.9%. However, large differences were observed across the different age groups, with olive oil contributing roughly 30% among children and adolescents, but nearly 50% in the older adult population. Other major contributors were sausages and meat derivatives in children and adolescents (contributing to a much lesser extent in the adult groups). As for total PUFA, oils and fats were also the main contributors, followed by meat and meat products and grains, whereas fish and shellfish accounted for 8.5% (25.9% of the total *n*-3 fatty acid intake). Olive oil was the highest individual contributor, from 25.9% for older adults to a much lower intake for children and adolescents (<15%). Differences were also seen according to age for meat and meat products (higher contributions from sausages and meat derivatives in the youngest groups) and for fish and shellfish (10.2% in older adults *vs*. 4.7% in children). Interestingly, meat ranked first in the older groups for *n*-6 fatty acid intake, whereas in the youngest groups, sausages and other meat products ranked first. Fish and shellfish were the main contributors to *n*-3 fatty acid intake only in older adults and adults and ranked second to meat among both children and adolescents.

## 4. Discussion

Assessing macronutrients’ distribution for the whole Spanish population, but also by sex and age, is important for health policy makers. Moreover, the detailed information on dietary sources for macronutrients is critical to better understand the strengths and weaknesses of diet quality. Despite that secular trends in energy intake remaining stable or even decreasing in many European countries, including Spain, the partitioning of the macronutrient distribution is worsening and somewhat moving away from the recommendations and traditional Mediterranean dietary pattern, as shown in the present ANIBES study. Although the negative changes affect all age groups and either males or females, those are less pronounced as age increase.

In the ANIBES study, overall protein intake was well above the upper recommended limit (15% Energy (E)) [21]. The dietary reference intake for total protein is about 0.8 g/kg body weight for adults, representing roughly 12% of energy intake [21]. The Spanish National Survey of Dietary Intake, the Encuesta Nacional de Ingesta Dietética España (ENIDE) study or trends observed in the Spain Food Consumption Survey (FCS) results were also equivalent [22,23]. Protein intake, regardless of sex or age group, was higher when compared to the updated dietary reference intakes for the Spanish population [20] (e.g., 54 g/day in adults and older adult men; 41 g/day in adults and older adult women) or those previously published by the European Food Safety Authority (EFSA) in 2012 and revised in 2015 [24]. In fact, only 10% of the ANIBES population (P10) would be within the recommended range for dietary protein intake. However, if we refer to the acceptable range proposed by the Institute of Medicine [25] for protein intake (10%–35% E), our results would be within the limits.

The importance of accurately defining the amount and quality of protein required to meet nutritional needs is well recognized at present, but describing how protein should be distributed (total and in terms of quality and sustainability) in food ingredients, whole foods or mixed diets is, as well.

EFSA has recently (2015) launched an updated Scientific Opinion on Dietary Reference Values (DRV) for protein, which includes typical intakes of protein for children and adolescents from 20 countries in Europe and from 24 countries in the case of the adults [24]. Differences in methodology and age classification make comparisons difficult. However, an overview shows that average protein intake ranges in children from 29 to 63 g/day, increasing to 61–116 g/day in adolescents, being higher in males in both age groups. In adults, average protein ranges from 67 to 114 g/day in men and from 59 to 102 g/day in women. Our findings from the ANIBES study show a mean intake of 74 g/day, also much higher in males and increasing with age, except for the elderly [24].

Data from food consumption surveys show that the actual mean protein intakes of adults in Europe are at, or more often above, the population reference intake (PRI) of 0.83 g/kg body weight per day [24]. In Europe, adult protein intakes at the upper end (90th–97.5th percentile) of the intake distributions have been reported to be between 17% E and 27% E. It is widely accepted that an excess of protein may counteract an adequate energy profile and a healthy dietary pattern. However, when consideration has been made to derive an upper level for protein, insufficient available evidence has been reported. The Institute of Medicine, in fact, reported very high protein intakes (up to 35% E) without negative effects [25]. The latter meaning that approximately an intake of twice the reference intakes should be considered safe in adults. This, however, must not be considered as adequate to achieve a healthy diet. In our Spanish ANIBES population, the protein intake distribution shows an inadequate high amount consumed, except for the elderly women. It should be necessary to keep in mind that when intakes are usually higher than 45% E, acute adverse outcomes may be expected.

The potential problem in association with long-term excess of protein would be, among others, how to maintain the nutrient density. On the other hand, it has been also postulated that a high increase in protein intake may favor a decrease in body weight and adiposity. However, these observations need to be well proven, since the mentioned effects may be also due to the concomitant modification of carbohydrate and/or fat intakes [26,27,28]. Most of the literature, however, has concluded that there is strong and consistent evidence that when energy intake is controlled, the macronutrient proportion of the diet is not directly related to weight loss [29]. Other potential adverse effects due to high protein intake are in relation to insulin sensitivity and glucose tolerance, with somewhat contradictory results [30,31,32]. The dual effect for protein intake may be seen also for its association with calcium and bone health: it is widely accepted that protein deficiency may increase the risk of bone fragility and fracture [33], whereas an increase in protein intake could also be associated with higher urinary calcium excretion [34]. Finally, and more importantly, intervention studies in humans have not shown remarkable effects of high protein intake on markers of bone health [35,36].

Although animal sources of proteins, including meat, poultry, seafood, milk and eggs, are the highest quality proteins, plant proteins may be also an excellent and complimentary source of proteins, mainly when mixed diets include combinations, such as legumes and grains, as is widely recommended. As collected by EFSA [24], in most European countries, the main contributor to the dietary protein intake is meat and meat products, followed by grains and grain-based products and milk and dairy products, contributing all together to about 75% of the protein intake. At that point (2011), meat and meat products represented a 32% contribution to dietary protein in Spain, much lower when compared to countries, such as Ireland, Poland or France. The trend seems to be stable in our country, since our ANIBES data show a similar percentage contribution. A marked and rapid increase for this food group has been shown in Spain in the last few decades [16,22], zooming out from the traditional Mediterranean diet where meat consumption used to be scarce. No significant changes in the last few years are observed in Spain for grains and grain-based products (by the way, the lowest contribution within Europe), whereas a decreasing trend in milk and dairy products and in fish and shellfish is clearly observed in recent years.

In the ENIDE study in Spain [22], most protein intake was also of animal origin (80%) and mainly from meats (31%), although fish (27%) was also a major contributor and much higher than its contribution in the present ANIBES study. It should be taken into consideration, however, that a sharp decline in fish consumption has taken place in recent years in Spain, which may compromise nutritional goals, especially among younger populations [23]. As mentioned, the FCS also showed a high protein intake (twice the PRIs) for the adult Spanish population in the last few years, mainly from meat and meat products (29.9%), followed by milk and milk products (16.6%), grains (16.5%) and fish and shellfish (11.3%) [23]. In conclusion, more efforts are needed to lower the excessive and nutritionally unnecessary amount of protein consumed by the Spanish population at present, but also to redistribute the animal/plant protein ratio.

Total fat intake should be higher than 15% E to provide the intake of the essential fatty acids and energy and to be able to facilitate the absorption of lipid-soluble vitamins. In general, with moderate physical activity, a 30% E from fat intake is recommended, and up to 35% in the case of a high physical activity level [25]. We show, however, that total fat intake in Spain is well above these recommendations and upper limits in some of the most sedentary societies, such as the Spanish society, nowadays. Interestingly, there is evidence that moderate fat intake (<35% E) is accompanied by reduced or adequate energy intake, and therefore, body weight control, moderate weight reduction and/or prevention of weight gain may be better achieved. However, EFSA has concluded that there is insufficient scientific evidence to define a lower threshold intake or tolerable upper intake level for total fat [37]. Presently, at the European level, but also from the World Health Organization (WHO) and Food Agriculture Organization (FAO), a lower boundary for the reference intake range of 20% E and an upper boundary of 35% E have been proposed [38]. In addition, it is well known that two main processes contribute to the development of ischemic heart disease: atherosclerosis and thrombosis. The type of dietary fat may contribute to both. Since some fatty acids have a greater role, consequently, the evaluation of updated dietary sources of fat may be a helpful tool to advise healthy dietary patterns to prevent cardiovascular diseases. Fats and oils are also important sources of essential fatty acids and some bioactive compounds of nutritional interest (e.g., polyphenols from olive oil). However, high-fat diets may decrease or impair insulin sensitivity and may be also positively associated with increased higher cardiovascular risk [39,40].

It is also important to understand how the different food and beverage groups and subgroups contribute to the quality of dietary fat, namely SFA, MUFA and PUFA, including *n*-6 and *n*-3 fatty acids, since dietary fat quality is markedly related to the etiology and/or prevention of different chronic degenerative diseases.

The intake of SFA has been generally recognized to be deleterious and therefore its determination is included in most of the diet quality indexes [41]. In contrast, higher consumption of MUFA and PUFA has been reported to be associated with reduced CVD risk. The minimum recommended level of total PUFA consumption to lower LDL-C and total cholesterol, increasing HDL-C concentrations in order to decrease the risk of CHD events, is 6% E. Our present data show that this level is easily achieved by the Spanish population as a whole. In contrast, higher risk of lipid peroxidation may occur with high (>11% E) PUFA consumption, although this does not seem to represent a risk in our population findings [25].

For infants in Europe, average intakes of SFA are usually higher than the recommended upper limit [37]. In adults, average SFA intakes according to the last available European Nutrition Health Report [13] vary between less than 9% E and 26% E, with the lowest values mostly observed in southern European countries. The SFA intake in the ANIBES study was also above the recommendations for all age groups and both sexes, a negative trend that is being observed in the last two decades [23]. However, no dietary reference intakes have been set at present, nor upper levels [37,38]. Even so, the WHO/FAO have recommended a maximum intake of 10% E for SFA [38], which also agrees with the Spanish Federation of Food, Nutrition and Dietetic Societies (FESNAD) Consensus Document on Dietary Fats and Oils for the Adult Spanish Population [41]. There is also evidence from dietary intervention studies that decreasing the intake of products rich in SFA and being replaced with products rich in *n*-6 PUFA (with no change in total fat intake) were effective in decreasing some cardiovascular events [42,43,44]. In the ANIBES study, >70% of SFA were obtained almost equally from meat and meat products, milk and dairy products and oils and fats. In children and adolescents, the highest contribution corresponded to the sausage and meat derivatives subgroup, followed by bakery and pastry; in adults and older adults, however, olive oil and meat ranked as the primary individual contributors. These trends, again, add difficulties at present to following the Mediterranean dietary patterns for the Spanish population, which is mainly of concern in the youngest.

Available combined data for MUFA intakes in Europe range between 8% E and 11% E in infants and mostly between 10% E and 13% E in children and adolescents [37]. In adults, the highest mean intake has been found in Greece (22%–23% E); in other European countries, average intakes vary between 11% E and 18% E. As for MUFA intake in the present ANIBES results, oils and fats were the major contributors, of which olive oil accounted for a high proportion. Undoubtedly, the latter is still one of the main strengths of the present Spanish diet, and all efforts are made to convince all age groups about its benefits for a better adherence to the Mediterranean diet. Despite this, large differences were observed across the different age groups, with olive oil contributing roughly 30% in children and adolescents, but nearly 50% in the older adult population. In our ANIBES population, MUFA intake was slightly higher in the older adult group and lower among children and adolescents, once again showing a better adherence to the principles of the traditional Mediterranean diet in the adult and senior populations. The most recent 2011 goals developed by the Spanish Society of Community Nutrition (SENC) [21] recommend that MUFA should contribute >20% E of total energy, whereas the FAO/WHO have recommended a MUFA intake of about 16%–19% E [38]. In contrast, an EFSA panel [37] proposed in 2010, however, not setting any dietary reference value for MUFA based on the following criteria: MUFA are synthesized by the body, with no known specific role in preventing or promoting diet-related diseases and are therefore not indispensable constituents of the diet. This assumption by EFSA, however, is rebuttable because MUFA are present in most tissues’ cells and have roles as key compounds to maintain membrane fluidity and diverse enzymatic activities [45]. In addition, MUFA may lower both total and LDL plasma cholesterol levels, potentially lowering also cardiovascular risk [46]. Moreover, in the Prevention with Mediterranean Diet (PREDIMED) intervention study [47], intake of virgin olive oil (MUFA at 22% E) was associated with much lower risk of CVD events and total mortality. Therefore, according to the PREDIMED findings, a MUFA intake target of 20%–25% E (with virgin olive oil as a main source) has been proposed. At the population level, the latter may be quite difficult to achieve.

In spite of the well-known metabolic effects of various dietary PUFA [43], EFSA has proposed not to formulate a dietary reference value for this fatty acid family [37]. Other organizations, such as the WHO/FAO in 2010 [38] and SENC (2011) [21], have suggested that PUFA should contribute 6%–10% E and 5% E, respectively. In the present ANIBES study, PUFA contributed roughly 6.6% E, with no sex or age differences, whereas *n*-3 PUFA intake expressed as the percentage of energy intake was 0.63% E for the ANIBES study population and increased with age. The WHO/FAO [38] have recommended a minimum intake for adults of 250 mg/day for *n*-3 long-chain PUFA and up to 2 g/day to help prevent CVD. For European children, average cis *n*-6 PUFA intakes in absolute amounts vary between approximately 5 g and 17 g per day (in the present ANIBES study, values were 12.0 ± 4.8 g/day in children and 12.6 ± 5.8 g/day in adolescents), with a much lower contribution for older adults (9.0 ± 5.3 g/day).

As for total PUFA food sources in the present study, as expected, oils and fats were also the primary contributors: olive oil was the greatest individual contributor, mainly in adults and seniors Interestingly, meat ranked first in the older groups for *n*-6 fatty acid intake, whereas sausages and other meat products ranked first among the youngest groups. The fish and shellfish food group was the main contributor to *n*-3 fatty acids only in older adults (31.4%) and adults (25.9%), but ranked second to meat and meat products in both children and adolescents. Once again, more efforts are necessary in children and adolescents to avoid the loss of some key principles of the healthy Mediterranean dietary pattern as derived from these ANIBES updated results.

WHO/FAO Expert Consultation [48,49,50] recommended initially that total carbohydrate (CHO) in the diet should provide 55%–75% E. Later, the same institutions suggested a new lower limit, 50% E, whereas EFSA in Europe proposed a range between 45% and 60% E. Finally, in Spain, the SENC recommend 50%–60% total energy [21]. The Spanish population, however, is well below the lower limit, which is considered a bad indicator of present diet quality.

Mean total carbohydrate intake was 185.4 ± 60.9 g/day (37.8 g/day min; 450.3 g/day max) and higher in men than in women. Higher total carbohydrate consumption was observed in the younger groups as compared to adults and older adults. Total sugar intake was also quantified: 76.3 ± 33.9 g/day (79.5 ± 36.6 g/day in men, 73.0 ± 30.6 g/day in women). Differences were also seen according to age group with significantly higher intakes in children and adolescents compared to those observed in adults and older adults.

In the latest EFSA Scientific Opinion on DRV for CHO and dietary fiber, data were presented for children and adolescents in 19 countries and for adults in 22 countries in Europe [50]. Even though there is a large diversity in the methodology used and age classification, as stated, the highest mean intakes were observed in the Czech Republic and Norway, whereas the lowest were found in Greece and Spain. As for fiber, average dietary intakes varied from 10 to 20 g/day in young children and from 15 to 33 g/day in adolescents, whereas in adults, it ranged from 15 to 30 g/day. Finally, for the elderly, most of the countries showed intakes from 20 to 25 g/day.

It is well known that dietary CHO shows a variety of physical, chemical and important physiological properties: control of body weight, diabetes, CVD, large bowel cancer, constipation and resistance to gut infection, caries and a low density of micronutrients, among others. In addition, to judge the quality of the diet, it is crucial to distinguish the different types of CHO and dietary sources, as shown in the present study; the latter since the main interest and concern are associated with the content of sugars (natural or added) and fiber, glycemic index, refined *vs*. whole-grains, the presence of fruits and vegetables or solid *vs*. liquid CHO [49,50].

CHO provide energy and can contribute to weight gain, being overweight and obesity when consumed in excess. Intervention studies have provided evidence that high fat (>35% E), low carbohydrate (<50% E) diets are associated with adverse short- and long-term effects on body weight, although the data are insufficient to define a lower threshold of intake for carbohydrates [51,52]. It is also known that frequent consumption of sugar-containing foods can increase the risk of dental caries [53]. However, the available data do not allow for setting an upper limit for the intake of added sugars on the basis of risk reduction for dental caries, which has not yet been proposed. Evidence relating a high intake of sugars (mainly added sugars) *versus* starches to weight gain is also inconsistent and controversial [54]. As a consequence, according to EFSA, the available data are insufficient to set an upper limit for added sugar intake [50], even though there is some evidence that high sugar intake (>20% E) may increase serum triglyceride and cholesterol concentrations and might adversely affect serum glucose and insulin levels, but this is still insufficient to set an upper limit for added sugar intake. The latter, however, does not exclude that food-based dietary guidelines and nutrition goals should take into account potential negative effects under certain conditions and reinforce the importance to limit sugar consumption. More strongly, a new WHO guideline [55] recommends that adults and children should reduce their daily intake of free sugars to less than 10% of their total energy intake according to their daily dietary energy reference intakes. A further reduction to below 5% has been proposed to potentially provide additional health benefits [55]. In this sense, the percentage of energy from sugars in our study was 17.0% E for the total population, significantly higher in females compared to males, and was more marked among the oldest participants, which shows that better educational campaigns and advice for the Spanish population is further needed. Paradoxically, secular trends of CHO intake in Spain show an inverse association with Spanish people affected by being overweight/obesity at all ages. Importantly, a diet high in fiber is usually considered also to have relatively low energy density, the promotion of satiety and a lower degree of weight gain. The percentage contribution of carbohydrates has steadily decreased since the 1960s in Spain. In that decade, the energy profile was in line with the recommendations [22]. It is remarkable also that when dietary fiber intake in Spain in the 1960s was much higher than the present data from the ANIBES study, the prevalence of excess weight was also quite lower. The current and increasing worsening is linked to a decline in the consumption of cereals and derivatives, legumes and pulses and potatoes. However, as expected, cereals and derivatives represent the highest contribution to total carbohydrates, followed by milk and derivatives. Whole-grain cereals, vegetables, legumes and fruits are the most recognized sources for dietary CHO due to their additional high content in fiber and low energy content. As derived from our present data, whole-grain cereals and legumes are consumed in lower amounts than recommended.

## 5. Conclusions

In conclusion, despite that secular trends in energy intake are remaining stable or even decreasing in many European countries, including Spain, the partitioning of the macronutrient distribution is worsening and clearly moving away from the recommendations.

The findings of the present ANIBES study are considered key to the future definition and revision of PRIs, dietary guidelines, nutritional goals in Spain and appropriate and specific targeted public campaigns. It seems that the new national strategy, including not only those responsible for the health policy, but also education stakeholders and involving gastronomy leaders, that is now being implemented may help to improve diet quality in Spain.

## Figures and Tables

**Table 1:** Daily nutrient intake and distribution in the Spanish ANIBES study population (9–75 years old).

Nutrients	Mean	Median	SD	SEM	P5	P10	P25	P50	P75	P90	P95	Min	Max
**Proteins (g)**	**74.5**	**71.8**	**22.4**	**0.5**	**43.9**	**48.2**	**59.2**	**71.8**	**87.0**	**103.9**	**112.3**	**28.2**	**352.5**
**Carbohydrates (g)**	**185.4**	**177.4**	**60.9**	**1.4**	**99.4**	**114.2**	**143.2**	**177.4**	**222.1**	**267.3**	**294.9**	**37.8**	**450.3**
Sugar (g)	76.3	71.5	33.9	0.8	30.0	37.3	52.5	71.5	96.2	122.9	136.7	6.7	263.6
**Lipids (g)**	**78.1**	**75.0**	**26.1**	**0.6**	**41.4**	**47.3**	**59.5**	**75.0**	**93.0**	**113.4**	**126.5**	**21.0**	**201.5**
SFA (g)	24.0	22.6	9.5	0.2	11.0	12.9	17.3	22.6	29.4	36.2	40.9	5.1	86.6
MUFA (g)	33.7	32.7	11.3	0.3	18.2	20.4	25.3	32.7	40.1	48.5	53.6	8.8	96.7
PUFA (g)	13.4	12.3	6.1	0.1	5.7	6.7	9.0	12.3	16.6	21.2	24.5	2.6	50.6
*n*-6 (g)	11.1	10.1	5.5	0.1	4.1	5.1	7.0	10.1	14.0	18.4	21.1	1.4	45.1
*n*-3 (g)	1.3	0.9	11.6	0.3	0.4	0.5	0.6	0.9	1.3	1.9	2.4	0.2	520.7
Cholesterol (mg)	315	298	137	3	136	162	215	298	389	492	557	11	1.584
**Fiber (g)**	**12.7**	**11.8**	**5.6**	**0.1**	**5.4**	**6.5**	**8.7**	**11.8**	**15.6**	**19.7**	**22.9**	**2.2**	**45.1**
**Alcohol (g)**	**5.4**	**0.0**	**10.6**	**0.2**	**0.0**	**0.0**	**0.0**	**0.0**	**6.8**	**17.3**	**26.2**	**0.0**	**110.8**
**Water (mL)**	**1626**	**1489**	**641**	**14**	**819**	**944**	**1173**	**1489**	**1960**	**2533**	**2842**	**368**	**5683**

SD: standard deviation; SEM: standar error; P: Percentil; SFA: saturated fatty acids; MUFA: monounsaturated fatty acids; PUFA: polyunsaturated fatty acids; *n*-6: omega-6 fatty acids; *n*-3: omega-3 fatty acids.

**Table 2:** Total daily nutrient intake by sex and age group in the Spanish ANIBES study population aged 9–75 years.

	Total			Children 9–12 Years			Adolescents 13–17 Years			Adults 18–64 Years			Elderly 65–75 Years		
	Total	Men	Women	Total	Men	Women	Total	Men	Women	Total	Men	Women	Total	Men	Women
*n*	2009	1013	996	213	126	87	211	137	74	1655	798	857	206	99	107
**ENERGY (kcal)**	**1810 (504)**	**1957 (531)**	1660 * (427)	**1960 (431)**	**2006 (456)**	1893 * (385)	**2018 (508)**	**2124 (515)**	1823 * (436)	**1816 (512)**	**1966 (543)**	1675 * (437)	**1618 (448)**	**1771 (485)**	1476 * (360)
**PROTEINS (g)**	**74.5 (22.4)**	**80.3 (24.9)**	68.5 * (17.7)	**77.6 (18.9)**	**80.6 (19)**	73.3 * (18.1)	**80.0 (21)**	**85.0 (21)**	70.6 * (17.7)	**74.8 (22.9)**	**81.0 (26)**	69.0 * (17.8)	**67.7 (21)**	**73.5 (23.9)**	62.4 * (16.3)
**CARBOHYDRATES (g)**	**185.4 (60.9)**	**200.0 (64.9)**	170.7 * (52.7)	**214.3 (57.1)**	**218.2 (61.1)**	**208.7 (50.7)**	**224.6 (67.5)**	**234.5 (70.0)**	206.1 * (58.8)	**184.0 (60.4)**	**198.7 (64.6)**	170.3 * (52.8)	**163.7 (53.4)**	**175.0 (59.7)**	153.3 * (44.7)
SUGAR (g)	76.3 (33.9)	79.5 (36.6)	73.0 * (30.6)	91.6 (33.3)	93.7 (35.3)	88.4 (30.1)	89.3 (35.1)	90.8 (37.2)	86.6 (31)	74.9 (33.8)	78.4 (36.7)	71.7 * (30.5)	73.0 (34.0)	74.2 (37.4)	71.8 (30.6)
**LIPIDS (g)**	**78.1 (26.1)**	**83.7 (27.2)**	72.4 * (23.6)	**85.1 (22.1)**	**87.3 (23.2)**	**82.1 (20.0)**	**85.9 (25.8)**	**90.9 (25.9)**	76.7 * (23.1)	**78.7 (26.5)**	**84.2 (27.8)**	73.6 * (24.2)	**67.4 (22.1)**	**73.2 (23.0)**	62.0 * (19.8)
SFA (g)	24.0 (9.5)	25.8 (10.0)	22.1 * (8.7)	28.7 (8.7)	29.6 (9.3)	27.5 (7.5)	28.3 (9.6)	30.0 (9.6)	25.2 * (9.0)	24.0 (9.6)	25.7 (10.1)	22.5 * (8.8)	19.3 (7.5)	20.8 (7.6)	17.9 * (7.1)
MUFA (g)	33.7 (11.3)	36.1 (11.9)	31.3 * (10.2)	34.9 (9.6)	35.8 (10.2)	33.6 (8.6)	35.1 (10.9)	37.3 (11.3)	31.2 * (8.9)	34.0 (11.6)	36.4 (12.3)	31.8 * (10.5)	30.6 (9.7)	33.1 (9.6)	28.3 * (9.2)
PUFA (g)	13.4 (6.1)	14.4 (6.5)	12.5 * (5.5)	14.1 (5.2)	14.2 (5.1)	14.0 (5.4)	14.7 (6.3)	15.4 (6.3)	13.4 * (6.2)	13.6 (6.1)	14.6 (6.5)	12.7 * (5.6)	11.4 (6.5)	12.6 (7.7)	10.3 * (5)
*n*-6 (g)	11.1 (5.5)	11.9 (5.8)	10.1 * (5)	12.0 (4.8)	12.1 (4.6)	11.9 (5.1)	12.6 (5.8)	13.2 (5.8)	11.5 * (5.7)	11.2 (5.5)	12.1 (5.9)	10.3 * (5.0)	9.0 (5.3)	9.9 (6.1)	8.3 * (4.4)
*n*-3 (g)	1.3 (11.6)	1.6 (16.3)	1.0 (0.7)	0.9 (0.5)	1.0 (0.5)	0.9 (0.5)	1.0 (0.6)	1.0 (0.6)	0.9 (0.5)	1.4 (12.8)	1.8 (18.4)	1.0 * (0.7)	1.1 (0.9)	1.4 (1.1)	0.9 * (0.5)
Cholesterol (mg)	315 (137)	345 (146)	284 * (121)	328(110)	347 (112)	299 * (102)	342 (139)	368 (139)	294 * (128)	316 (137)	347 (144)	287 * (122)	296 (153)	320 (174)	273 * (128)
**FIBRE (g)**	**12.7 (5.6)**	**13.1 (6.1)**	12.2 * (5.2)	**11.8 (4.3)**	**11.5 (4.0)**	**12.2 (4.6)**	**11.8 (4.7)**	**12.1 (4.8)**	**11.2 (4.6)**	**12.6 (5.7)**	**13.1 (6.1)**	12.1 * (5.2)	**14.6 (6.8)**	**15.7 (7.7)**	13.6 * (5.6)
**ALCOHOL (g)**	**5.4 (10.6)**	**7.3 (12.8)**	3.5 * (7.3)	**0.0 (0)**	**0.0 (0)**	**0.0 (0)**	**0.1 (0.6)**	**0.0 (0.4)**	**0.1 (0.8)**	**6.1 (11.1)**	**8.3 (13.3)**	4.0 * (8.0)	**7.0 (12.6)**	**10.8 (14.8)**	3.5 * (8.7)
**WATER (mL)**	**1626 (641)**	**1666 (679)**	1585 * (596)	**1392 (484)**	**1432 (514)**	**1335 (434)**	**1336 (464)**	**1391 (511)**	1236 * (345)	**1663 (661)**	**1722 (703)**	1608 * (614)	**1583 (539)**	**1586 (575)**	**1580 (506)**

Results are expressed as the mean ± the standard deviation (in brackets); * denotes statistical difference (*p* ≤ 005) by sex; SFA: saturated fatty acids; MUFA: monounsaturated fatty acids; PUFA: polyunsaturated fatty acids; *n*-6: omega-6 fatty acids; *n*-3: omega-3 fatty acids.

**Table 3:** Dietary sources of nutrients (%) from food groups/subgroups in the Spanish ANIBES study population aged 9–75 years (*n* = 2009).

	Total (Aged 9–75 Years)														
	Total Intake (Weight)	Energy	Water	Proteins	Lipids	SFA	MUFA	PUFA	*n*-6	*n*-3	Carbohydrates	Sugar	Fiber	Cholesterol	Alcohol
	%	%	%	%	%	%	%	%	%	%	%	%	%	%	%
**Grains**	**7.93**	**27.40**	**2.25**	**17.38**	**10.35**	**10.75**	**7.46**	**14.68**	**9.82**	**5.24**	**48.97**	**11.98**	**39.90**	**8.60**	**-**
Grains and flours	1.22	4.47	0.17	2.61	0.44	0.30	0.24	1.07	0.71	0.17	8.75	0.25	3.40	-	-
Bread	3.90	11.57	1.62	8.35	1.84	1.41	1.20	3.89	2.42	1.36	23.37	2.84	20.72	0.00	-
Breakfast cereals and cereal bars	0.23	1.00	0.02	0.56	0.23	0.30	-	-	-	-	1.83	1.29	1.57	-	-
Pasta	0.98	3.56	0.12	3.09	0.50	0.24	0.17	1.33	0.88	0.41	6.43	0.91	6.21	0.09	-
Bakery and pastry	1.60	6.80	0.32	2.77	7.34	8.49	5.84	8.39	5.81	3.30	8.58	6.69	8.00	8.52	-
**Vegetables**	**8.82**	**4.02**	**8.94**	**3.79**	**0.59**	**0.33**	**0.17**	**1.55**	**0.34**	**1.26**	**7.66**	**7.28**	**23.67**	**0.01**	**-**
**Fruits**	**7.16**	**4.75**	**5.97**	**1.90**	**1.86**	**1.06**	**1.59**	**3.13**	**1.44**	**1.13**	**8.21**	**16.78**	**17.18**	**-**	**-**
**Oils and fats**	**1.29**	**12.29**	**0.05**	**0.03**	**32.19**	**21.39**	**42.37**	**33.02**	**19.48**	**7.58**	**0.01**	**0.02**	**-**	**2.45**	**-**
Olive oil	0.91	9.22	0.00	-	24.41	15.01	36.96	18.46	9.84	4.14	-	-	-	-	-
Other oils	0.19	1.71	-	-	4.41	2.03	3.06	11.49	6.59	0.13	-	-	-	-	-
Butter, margarine and shortening	0.19	1.37	0.05	0.03	3.38	4.35	2.35	3.07	3.06	3.30	0.01	0.02	-	2.45	-
**Milk and dairy products**	**13.38**	**11.81**	**14.95**	**17.17**	**13.48**	**23.67**	**8.87**	**2.83**	**10.00**	**13.10**	**9.90**	**23.26**	**0.40**	**14.69**	**-**
Milks	9.10	4.98	10.82	8.05	4.62	7.94	2.99	0.77	4.21	7.34	5.02	12.71	-	5.78	-
Cheeses	0.90	2.99	0.59	5.34	5.34	9.33	3.42	1.25	3.48	3.12	0.21	0.55	-	5.04	-
Yogurt and fermented milk	2.37	2.37	2.54	2.86	1.77	3.45	1.22	0.36	1.69	2.01	3.09	6.82	0.30	1.41	-
Other dairy products	1.01	1.47	0.99	0.92	1.76	2.96	1.23	0.45	0.62	0.63	1.58	3.17	0.10	2.46	-
**Fish and shellfish**	**3.03**	**3.55**	**2.21**	**10.63**	**4.21**	**3.10**	**2.94**	**8.53**	**9.89**	**25.88**	**0.07**	**0.03**	**-**	**12.16**	**-**
**Meat and meat products**	**7.79**	**15.16**	**6.26**	**33.14**	**22.52**	**25.74**	**22.50**	**20.33**	**38.70**	**38.29**	**0.30**	**0.58**	**-**	**35.97**	**-**
Meat	5.54	9.25	4.54	21.83	12.74	14.60	12.79	11.29	21.94	25.71	0.00	0.02	-	24.20	-
Sausages and other meat products	2.16	5.79	1.64	10.95	9.65	10.99	9.63	8.96	16.50	12.32	0.29	0.56	-	11.10	-
Viscera and spoils	0.09	0.11	0.09	0.35	0.13	0.15	0.08	0.07	0.26	0.26	0.01	-	-	0.68	-
**Eggs**	**1.53**	**2.20**	**1.37**	**4.68**	**3.76**	**3.92**	**3.09**	**3.83**	**2.11**	**1.70**	**0.00**	**0.01**	**-**	**20.81**	**-**
**Pulses**	**0.73**	**2.25**	**0.23**	**3.32**	**0.49**	**0.12**	**0.31**	**1.27**	**0.55**	**0.04**	**3.24**	**0.57**	**9.39**	**-**	**-**
**Sugars and sweets**	**0.83**	**3.34**	**0.12**	**0.84**	**1.40**	**2.14**	**1.19**	**0.90**	**0.54**	**0.62**	**6.52**	**15.13**	**0.68**	**0.25**	**-**
Sugar	0.32	1.37	0.01	0.00	-	-	-	-	-	-	3.35	8.18	-	-	-
Chocolates	0.37	1.54	0.06	0.82	1.38	2.13	1.16	0.87	0.52	0.61	2.17	4.74	0.45	0.24	-
Jams and other	0.11	0.33	0.05	0.01	-	-	-	-	-	-	0.78	1.77	0.19	-	-
Other sweets	0.03	0.10	0.01	0.02	0.02	0.01	0.03	0.03	0.01	0.00	0.21	0.44	0.04	0.01	-
**Appetizers**	**0.26**	**0.79**	**0.14**	**0.32**	**1.11**	**1.04**	**1.40**	**0.88**	**0.47**	**0.16**	**0.74**	**0.15**	**1.45**	**0.08**	**-**
**Ready-to-eat meals**	**3.66**	**4.21**	**3.62**	**4.60**	**4.48**	**4.83**	**4.15**	**4.41**	**3.83**	**3.23**	**4.28**	**1.30**	**4.50**	**2.77**	**-**
**Sauces and condiments**	**0.70**	**1.57**	**0.64**	**0.50**	**3.16**	**1.70**	**3.79**	**3.84**	**2.83**	**1.75**	**0.36**	**0.70**	**2.12**	**1.96**	**-**
**Non-alcoholic beverage**	**38.40**	**3.95**	**47.71**	**1.18**	**0.34**	**0.18**	**0.17**	**0.75**	**-**	**-**	**8.36**	**18.57**	**0.53**	**-**	**-**
Water	24.01	-	30.04	-	-	-	-	-	-	-	-	-	-	-	-
Coffee and infusions	4.27	0.19	5.46	0.46	0.00	-	-	-	-	-	0.35	0.94	-	-	-
Sugar soft drinks	4.78	2.03	5.74	-	-	-	-	-	-	-	4.62	10.01	-	-	-
Non-sweetened soft drinks	1.79	0.02	2.28	0.00	0.05	-	-	-	-	-	0.02	0.08	-	-	-
Sports drinks	0.20	0.07	0.24	-	-	-	-	-	-	-	0.17	0.35	-	-	-
Energy drinks	0.06	0.04	0.07	-	-	-	-	-	-	-	0.08	0.16	-	-	-
Juices and nectars	2.59	1.34	3.05	0.31	0.01	-	-	-	-	-	2.91	6.55	0.49	-	-
Other drinks	0.68	0.26	0.82	0.41	0.28	0.18	0.17	0.75	-	-	0.22	0.49	0.05	-	-
**Alcoholic beverages**	**4.47**	**2.62**	**5.55**	**0.36**	**-**	**-**	**-**	**-**	**-**	**-**	**1.34**	**3.60**	**-**	**-**	**100.00**
Low alcohol content beverages	4.39	2.39	5.45u	0.36	-	-	-	-	-	-	1.29	3.51	-	-	93.45
High alcohol content beverages	0.09	0.23	0.10	-	-	-	-	-	-	-	0.04	0.09	-	-	6.55
**Supplements and meal replacement**	**0.01**	**0.09**	**-**	**0.17**	**0.06**	**0.04**	**0.01**	**0.06**	**-**	**0.03**	**0.05**	**0.04**	**0.13**	**0.03**	**-**

**Table 4:** Dietary sources of nutrients (%) from food groups/subgroups in the Spanish ANIBES study population: children aged 9–12 years (*n* = 213).

	Children (Aged 9–12 Years)														
	Total Intake (Weight)	Energy	Water	Proteins	Lipids	SFA	MUFA	PUFA	*n*-6	*n*-3	Carbohydrates	Sugar	Fiber	Cholesterol	Alcohol
	%	%	%	%	%	%	%	%	%	%	%	%	%	%	%
**Grains**	9.78	29.57	2.65	18.26	12.30	13.29	9.14	16.13	10.54	5.86	48.99	14.82	46.88	10.45	-
Grains and flours	1.30	3.88	0.18	2.27	0.32	0.20	0.17	0.83	0.59	0.16	7.35	0.21	3.11	-	-
Bread	4.39	10.99	1.90	8.01	1.93	1.35	1.48	4.04	2.40	1.48	20.50	2.20	20.78	-	-
Breakfast cereals and cereal bars	0.44	1.57	0.04	0.77	0.34	0.58	-	-	-	-	2.82	2.36	2.64	-	-
Pasta	1.22	3.96	0.13	3.60	0.51	0.20	0.18	1.49	0.85	0.49	6.85	0.73	7.54	0.09	-
Bakery and pastry	2.42	9.17	0.40	3.61	9.19	10.98	7.31	9.78	6.69	3.72	11.48	9.32	12.82	10.35	-
**Vegetables**	6.78	2.96	7.04	2.57	0.34	0.18	0.07	0.93	0.25	0.90	5.57	3.61	17.52	0.00	-
**Fruits**	5.32	2.89	4.54	1.22	1.06	0.56	0.99	2.02	0.94	0.74	4.78	9.80	13.03	-	-
**Oils and fats**	1.31	10.35	0.05	0.02	26.67	15.72	35.80	31.04	17.68	6.89	0.01	0.02	-	2.22	-
Olive oil	0.86	7.09	-	-	18.45	9.79	29.86	14.40	7.17	3.44	-	-	-	-	-
Other oils	0.24	1.99	-	-	5.15	2.14	3.73	13.82	7.80	0.15	-	-	-	-	-
Butter, margarine and shortening	0.21	1.27	0.05	0.02	3.07	3.79	2.21	2.82	2.71	3.31	0.01	0.02	-	2.22	-
**Milk and dairy products**	20.66	15.41	23.84	20.77	17.64	28.76	12.06	3.68	11.96	15.63	12.89	29.87	0.52	19.30	-
Milks	13.66	6.71	16.76	10.25	7.20	11.30	4.82	1.28	5.51	9.38	5.74	13.86	-	8.61	-
Cheeses	0.84	2.53	0.53	4.62	4.57	7.38	3.11	1.13	2.57	2.68	0.12	0.32	-	4.48	-
Yogurt and fermented milk	3.36	3.06	3.65	3.43	2.51	4.61	1.78	0.46	1.87	2.45	3.66	8.70	0.06	1.63	-
Other dairy products	2.79	3.12	2.90	2.47	3.36	5.46	2.35	0.82	2.01	1.11	3.36	7.00	0.47	4.58	-
**Fish and shellfish**	2.38	2.13	1.92	7.24	2.22	1.43	1.58	4.68	6.92	20.09	0.02	0.01	-	8.19	-
**Meat and meat products**	8.57	14.90	7.09	32.50	22.96	24.28	24.21	21.56	39.81	41.01	0.39	0.67	-	34.24	-
Meat	5.67	7.98	4.83	19.79	11.16	11.92	11.83	9.98	18.96	24.61	0.01	0.02	-	21.42	-
Sausages and other meat products	2.89	6.89	2.25	12.65	11.77	12.34	12.36	11.57	20.77	16.40	0.38	0.65	-	12.67	-
Viscera and spoils	0.02	0.03	0.02	0.06	0.03	0.02	0.01	0.01	0.08	0.01	0.00	-	-	0.15	-
**Eggs**	1.55	1.90	1.42	4.13	3.21	2.88	2.78	3.45	1.76	1.57	0.00	0.00	-	19.76	-
**Pulses**	0.72	1.97	0.21	3.02	0.40	0.09	0.26	1.17	0.42	0.02	2.64	0.32	9.02	-	-
**Sugars and sweets**	1.41	5.07	0.25	2.20	3.60	4.57	3.29	3.18	1.85	2.08	7.79	17.18	1.02	0.48	-
Sugar	0.14	0.52	0.00	0.00	-	-	-	-	-	-	1.19	2.57	-	-	-
Chocolates	1.10	4.15	0.18	2.17	3.58	4.55	3.27	3.18	1.85	2.08	5.76	12.73	0.91	0.48	-
Jams and other	0.08	0.18	0.03	0.00	-	-	-	-	-	-	0.41	0.95	0.10	-	-
Other sweets	0.09	0.22	0.04	0.02	0.01	0.02	0.01	0.01	-	-	0.44	0.93	0.01	-	-
**Appetizers**	0.29	1.08	0.06	0.56	1.32	1.28	1.53	1.35	0.40	0.09	1.06	0.12	2.05	0.09	-
**Ready-to-eat meals**	4.79	5.53	4.73	6.32	5.80	5.75	5.42	6.44	4.67	3.76	5.22	1.32	6.27	4.05	-
**Sauces and condiments**	0.94	1.36	0.96	0.61	2.44	1.21	2.86	4.32	2.80	1.36	0.48	0.82	2.86	1.22	-
**Non-alcoholic beverage**	35.49	4.87	45.23	0.57	0.04	0.01	0.01	0.06	-	-	10.16	21.44	0.82	-	-
Water	22.92	-	29.93	-	-	-	-	-	-	-	-	-	-	-	-
Coffee and infusions	0.26	0.00	0.35	0.02	-	-	-	-	-	-	0.01	0.02	-	-	-
Sugar soft drinks	4.83	1.86	5.89	-	-	-	-	-	-	-	4.09	8.52	-	-	-
Non-sweetened soft drinks	0.95	0.01	1.25	0.00	0.01	-	-	-	-	-	0.00	0.01	-	-	-
Sports drinks	0.46	0.15	0.57	-	-	-	-	-	-	-	0.30	0.63	-	-	-
Energy drinks	0.02	0.00	0.03	-	-	-	-	-	-	-	-	-	-	-	-
Juices and nectars	5.99	2.83	7.13	0.51	0.01	-	-	-	-	-	5.76	12.24	0.82	-	-
Other drinks	0.06	0.02	0.07	0.05	0.02	0.01	0.01	0.06	-	-	0.00	0.01	-	-	-
**Alcoholic beverages**	0.00	0.00	0.00	-	-	-	-	-	-	-	0.00	0.00	-	-	100.00
Low alcohol content beverages	-	-	-	-	-	-	-	-	-	-	-	-	-	-	-
High alcohol content beverages	0.00	0.00	0.00	-	-	-	-	-	-	-	0.00	0.00	-	-	100.00
**Supplements and meal replacement**	**0.00**	**-**	**-**	**-**	**-**	**-**	**-**	**-**	**-**	**-**	**-**	**-**	**-**	**-**	**-**

**Table 5:** Dietary sources of nutrients (%) from food groups/subgroups in the Spanish ANIBES study population: adolescents aged 13–17 years (*n* = 211).

	Adolescents (Aged 13–17 Years)														
	Intake (Weight)	Energy	Water	Proteins	Lipids	SFA	MUFA	PUFA	*n*-6	*n*-3	Carbohydrates	Sugar	Fiber	Cholesterol	Alcohol
	%	%	%	%	%	%	%	%	%	%	%	%	%	%	%
**Grains**	10.82	30.69	3.01	19.07	12.73	13.08	9.31	17.81	10.80	5.94	50.80	14.96	48.84	10.86	-
Grains and flours	1.53	4.61	0.19	2.69	0.39	0.25	0.22	0.93	0.68	0.19	8.57	0.23	3.21	-	-
Bread	4.82	11.35	2.12	8.32	1.98	1.43	1.50	4.07	2.34	1.41	21.37	2.59	22.78	-	-
Breakfast cereals and cereal bars	0.57	1.94	0.05	0.84	0.47	0.78	-	-	-	-	3.47	3.13	3.04	-	-
Pasta	1.52	4.47	0.19	3.92	0.63	0.26	0.22	1.72	1.02	0.55	7.74	1.17	8.82	0.08	-
Bakery and pastry	2.37	8.32	0.46	3.30	9.26	10.36	7.37	11.08	6.77	3.79	9.65	7.84	10.98	10.77	-
**Vegetables**	6.82	2.95	7.18	2.61	0.44	0.27	0.19	1.08	0.23	0.91	5.38	3.79	18.31	0.01	-
**Fruits**	4.25	2.32	3.68	1.02	0.86	0.51	0.81	1.51	0.70	0.68	3.73	7.99	10.11	-	-
**Oils and fats**	1.32	9.75	0.05	0.02	25.39	15.68	33.67	28.80	17.07	6.72	0.01	0.02	-	2.13	-
Olive oil	0.85	6.62	0.00	-	17.43	9.76	27.92	13.42	7.06	3.31	-	-	-	-	-
Other oils	0.26	1.91	-	-	4.92	2.12	3.54	12.96	7.33	0.15	-	-	-	-	-
Butter, margarine and shortening	0.21	1.22	0.05	0.02	3.04	3.80	2.21	2.42	2.68	3.26	0.01	0.02	-	2.13	-
**Milk and dairy products**	17.62	12.60	20.68	17.93	15.13	25.09	10.39	3.07	10.55	14.69	9.73	24.03	0.66	16.57	-
Milks	12.37	5.78	15.41	8.91	6.22	9.88	4.30	1.07	5.25	9.05	4.98	13.20	-	7.43	-
Cheeses	1.00	2.87	0.63	5.26	5.21	8.77	3.45	1.21	3.16	3.63	0.13	0.40	-	5.04	-
Yogurt and fermented milk	2.30	2.00	2.54	2.23	1.60	2.97	1.14	0.27	1.05	1.39	2.47	5.78	0.26	1.20	-
Other dairy products	1.94	1.94	2.10	1.53	2.11	3.46	1.50	0.52	1.08	0.61	2.15	4.64	0.40	2.91	-
**Fish and shellfish**	2.29	2.09	1.84	6.33	2.59	1.82	1.98	5.12	6.08	17.68	0.02	0.03	-	7.21	-
**Meat and meat products**	9.49	15.95	7.98	34.62	24.43	26.30	25.30	22.74	41.27	43.00	0.36	0.69	-	36.07	-
Meat	6.54	9.05	5.69	22.06	12.56	13.54	13.07	11.41	20.97	27.23	0.01	0.03	-	23.17	-
Sausages and other meat products	2.93	6.88	2.26	12.47	11.86	12.74	12.22	11.33	20.22	15.72	0.35	0.66	-	12.75	-
Viscera and spoils	0.02	0.01	0.02	0.08	0.01	0.01	0.01	0.00	0.07	0.05	-	-	-	0.14	-
**Eggs**	1.85	2.06	1.86	4.50	3.46	3.26	3.08	3.52	1.82	1.74	0.00	0.02	-	20.16	-
**Pulses**	0.73	1.95	0.18	2.90	0.42	0.11	0.26	1.07	0.38	0.03	2.77	0.55	8.80	-	-
**Sugars and sweets**	1.26	4.36	0.19	1.85	3.03	4.03	2.77	2.41	1.36	1.60	6.82	16.33	0.87	0.31	-
Sugar	0.22	0.70	0.01	0.00	-	-	-	-	-	-	1.61	3.85	-	-	-
Chocolates	0.92	3.32	0.15	1.80	2.99	4.01	2.71	2.34	1.35	1.59	4.53	10.85	0.69	0.30	-
Jams and other	0.06	0.14	0.03	0.00	-	-	-	-	-	-	0.31	0.85	0.08	-	-
Other sweets	0.07	0.20	0.01	0.04	0.04	0.01	0.06	0.06	0.01	0.00	0.37	0.78	0.09	0.01	-
**Appetizers**	0.33	0.99	0.09	0.47	1.17	1.27	1.38	0.95	0.43	0.11	1.06	0.12	1.29	-	-
**Ready-to-eat meals**	5.55	6.51	5.27	7.46	6.87	6.90	6.63	7.17	6.09	4.68	6.21	1.69	7.43	4.42	-
**Sauces and condiments**	1.02	1.64	1.06	0.57	3.33	1.64	4.12	4.53	3.22	2.23	0.46	1.00	2.75	2.11	-
**Non-alcoholic beverage**	36.55	6.08	46.81	0.61	0.15	0.06	0.10	0.24	-	-	12.60	28.73	0.91	-	-
Water	19.98	-	26.18	-	-	-	-	-	-	-	-	-	-	-	-
Coffee and infusions	0.71	0.02	0.98	0.05	0.00	-	-	-	-	-	0.04	0.14	-	-	-
Sugar soft drinks	8.78	3.37	10.87	-	-	-	-	-	-	-	7.27	16.41	-	-	-
Non-sweetened soft drinks	1.19	0.01	1.63	0.00	0.02	-	-	-	-	-	0.01	0.03	-	-	-
Sports drinks	0.11	0.03	0.13	-	-	-	-	-	-	-	0.10	0.17	-	-	-
Energy drinks	0.17	0.09	0.20	-	-	-	-	-	-	-	0.16	0.37	-	-	-
Juices and nectars	5.38	2.46	6.51	0.42	0.02	-	-	-	-	-	4.96	11.46	0.85	-	-
Other drinks	0.24	0.10	0.30	0.13	0.11	0.06	0.10	0.24	-	-	0.07	0.14	0.06	-	-
**Alcoholic beverages**	0.10	0.04	0.13	0.01	-	-	-	-	-	-	0.02	0.05	-	-	100.00
Low alcohol content beverage	0.10	0.04	0.13	0.01	-	-	-	-	-	-	0.02	0.05	-	-	100.00
High alcohol content beverage	-	-	-	-	-	-	-	-	-	-	-	-	-	-	-
**Supplements and meal replacement**	0.01	0.02	-	0.02	0.00	0.00	0.00	0.00	-	-	0.03	0.01	0.03	0.01	-

**Table 6:** Dietary sources of nutrients (%) from food groups/subgroups in the Spanish ANIBES study population: adults aged 18–64 years (*n* = 1655).

	Adults (Aged 18–64 Years)														
	Total Intake (Weight)	Energy	Water	Proteins	Lipids	SFA	MUFA	PUFA	*n*-6	*n*-3	Carbohydrates	Sugar	Fiber	Cholesterol	Alcohol
	%	%	%	%	%	%	%	%	%	%	%	%	%	%	%
**Grains**	7.79	27.27	2.22	17.35	10.21	10.56	7.34	14.53	9.72	5.24	49.10	11.92	40.00	8.36	-
Grains and flours	1.21	4.51	0.18	2.64	0.44	0.31	0.24	1.07	0.71	0.18	8.88	0.27	3.46	-	-
Bread	3.85	11.57	1.59	8.35	1.85	1.42	1.21	3.87	2.42	1.35	23.47	2.93	21.02	0.00	-
Breakfast cereals and cereal bars	0.21	0.96	0.02	0.55	0.22	0.25	-	-	-	-	1.75	1.20	1.53	-	-
Pasta	0.98	3.60	0.13	3.12	0.52	0.26	0.19	1.32	0.87	0.42	6.55	0.94	6.28	0.11	-
Bakery and pastry	1.54	6.63	0.31	2.70	7.17	8.31	5.70	8.26	5.73	3.29	8.46	6.58	7.71	8.25	-
**Vegetables**	8.91	4.08	9.01	3.86	0.60	0.33	0.18	1.54	0.35	1.28	7.82	7.60	24.17	0.01	-
**Fruits**	6.74	4.60	5.59	1.88	1.93	1.09	1.67	3.24	1.51	1.16	7.83	16.18	16.60	-	-
**Oils and fats**	1.28	12.25	0.04	0.03	32.01	21.30	42.03	32.96	19.44	7.51	0.01	0.02	-	2.47	-
Olive oil	0.90	9.12	0.00	-	24.12	14.84	36.57	18.06	9.59	4.06	-	-	-	-	-
Other oils	0.20	1.78	-	-	4.57	2.13	3.17	11.88	6.81	0.14	-	-	-	-	-
Butter, margarine and shortening	0.18	1.35	0.04	0.03	3.32	4.33	2.30	3.02	3.04	3.31	0.01	0.02	-	2.47	-
**Milk and dairy products**	12.68	11.57	14.04	16.85	13.35	23.51	8.80	2.80	9.85	12.99	9.62	22.77	0.39	14.54	-
Milks	8.57	4.74	10.15	7.71	4.34	7.50	2.82	0.72	3.96	7.15	4.88	12.52	-	5.44	-
Cheeses	0.93	3.12	0.61	5.55	5.57	9.77	3.57	1.29	3.66	3.24	0.22	0.58	-	5.29	-
Yogurt and fermented milk	2.23	2.26	2.38	2.74	1.67	3.26	1.16	0.34	1.65	1.94	2.97	6.60	0.32	1.33	-
Other dairy products	0.94	1.45	0.90	0.86	1.77	2.98	1.25	0.45	0.59	0.67	1.54	3.06	0.07	2.48	-
**Fish and shellfish**	3.00	3.58	2.17	10.61	4.30	3.14	3.06	8.72	9.90	25.92	0.08	0.02	-	12.09	-
**Meat and meat products**	7.82	15.34	6.28	33.51	22.62	25.96	22.61	20.33	38.92	38.37	0.30	0.59	-	36.39	-
Meat	5.60	9.42	4.58	22.17	12.88	14.78	12.93	11.38	22.16	25.77	0.01	0.02	-	24.58	-
Sausages and other meat products	2.14	5.80	1.62	10.97	9.60	11.03	9.59	8.87	16.50	12.32	0.29	0.58	-	11.10	-
Viscera and spoils	0.09	0.12	0.09	0.36	0.13	0.15	0.08	0.08	0.27	0.28	0.01	-	-	0.71	-
**Eggs**	1.50	2.19	1.34	4.65	3.73	3.92	3.07	3.73	2.08	1.70	0.00	0.01	-	20.65	-
**Pulses**	0.72	2.22	0.24	3.28	0.49	0.12	0.30	1.26	0.53	0.04	3.20	0.59	9.28	-	-
**Sugars and sweets**	0.80	3.28	0.11	0.77	1.27	2.03	1.06	0.76	0.45	0.54	6.55	15.34	0.67	0.23	-
Sugar	0.34	1.48	0.01	0.00	-	-	-	-	-	-	3.63	8.95	-	-	-
Chocolates	0.32	1.39	0.05	0.74	1.25	2.02	1.02	0.71	0.43	0.54	1.98	4.28	0.44	0.22	-
Jams and other	0.11	0.31	0.05	0.01	-	-	-	-	-	-	0.74	1.70	0.18	-	-
Other sweets	0.03	0.10	0.00	0.02	0.03	0.01	0.04	0.05	0.02	0.00	0.20	0.41	0.05	0.01	-
**Appetizers**	0.27	0.81	0.14	0.32	1.16	1.07	1.47	0.90	0.49	0.17	0.76	0.15	1.53	0.08	-
**Ready-to-eat meals**	3.46	4.22	3.36	4.53	4.48	4.88	4.16	4.25	3.77	3.13	4.38	1.32	4.50	2.76	-
**Sauces and condiments**	0.71	1.67	0.63	0.50	3.38	1.82	4.03	4.01	3.01	1.91	0.36	0.72	2.18	2.11	-
**Non-alcoholic beverage**	39.32	3.93	48.67	1.26	0.40	0.21	0.20	0.90	-	-	8.37	18.62	0.49	-	-
Water	24.48	-	30.46	-	-	-	-	-	-	-	-	-	-	-	-
Coffee and infusions	4.63	0.20	5.91	0.48	0.00	-	-	-	-	-	0.37	1.01	-	-	-
Sugar soft drinks	4.78	2.06	5.70	-	-	-	-	-	-	-	4.72	10.22	-	-	-
Non-sweetened soft drinks	2.03	0.03	2.58	0.01	0.06	-	-	-	-	-	0.02	0.09	-	-	-
Sports drinks	0.24	0.09	0.28	-	-	-	-	-	-	-	0.20	0.42	-	-	-
Energy drinks	0.06	0.04	0.06	-	-	-	-	-	-	-	0.08	0.16	-	-	-
Juices and nectars	2.36	1.23	2.76	0.30	0.01	-	-	-	-	-	2.73	6.17	0.45	-	-
Other drinks	0.75	0.30	0.91	0.48	0.33	0.21	0.20	0.90	-	-	0.25	0.56	0.04	-	-
**Alcoholic beverages**	4.99	2.89	6.17	0.42	-	-	-	-	-	-	1.54	4.10	-	-	100.00
Low alcohol content beverages	4.90	2.63	6.06	0.42	-	-	-	-	-	-	1.49	3.99	-	-	92.95
High alcohol content beverages	0.10	0.26	0.11	-	-	-	-	-	-	-	0.05	0.11	-	-	7.05
**Supplements and meal replacement**	0.02	0.10	-	0.19	0.07	0.05	0.01	0.07	-	0.03	0.06	0.05	0.14	0.04	-

**Table 7:** Dietary sources of nutrients (%) from food groups/subgroups in the Spanish ANIBES study population: seniors aged 65–75 years (*n* = 206).

	SENIORS (Aged 65–75 Years)														
	Total Intake (Weight)	Energy	Water	Proteins	Lipids	SFA	MUFA	PUFA	*n*-6	*n*-3	Carbohydrates	Sugar	Fiber	Cholesterol	Alcohol
	%	%	%	%	%	%	%	%	%	%	%	%	%	%	%
**Grains**	6.74	25.59	1.95	16.38	9.24	9.56	6.46	13.69	9.84	4.60	46.89	10.13	31.39	8.23	-
Grains and flours	1.00	3.97	0.13	2.31	0.43	0.31	0.24	1.12	0.76	0.13	7.66	0.19	2.81	-	-
Bread	3.67	12.15	1.50	8.87	1.69	1.35	0.87	3.93	2.59	1.27	25.17	2.69	17.32	-	-
Breakfast cereals and cereal bars	0.14	0.75	0.01	0.47	0.14	0.11	-	-	-	-	1.45	0.67	0.86	-	-
Pasta	0.57	2.43	0.05	2.08	0.32	0.15	0.08	1.01	0.77	0.26	4.47	0.56	3.70	-	-
Bakery and pastry	1.36	6.29	0.26	2.65	6.67	7.64	5.27	7.63	5.73	2.93	8.14	6.02	6.70	8.23	-
**Vegetables**	10.49	4.86	10.64	4.78	0.72	0.45	0.11	2.16	0.45	1.62	8.89	8.38	25.48	0.01	-
**Fruits**	13.13	8.53	11.11	3.18	2.90	1.69	2.04	5.18	2.56	1.97	15.32	29.45	28.17	-	-
**Oils and fats**	1.41	14.86	0.06	0.04	39.91	28.31	53.12	35.90	21.12	9.26	0.01	0.03	-	2.86	-
Olive oil	1.10	12.24	-	-	33.22	21.64	48.40	25.99	13.93	5.43	-	-	-	-	-
Other oils	0.08	0.77	-	-	2.09	1.04	1.44	5.45	3.23	0.06	-	-	-	-	-
Butter, margarine and shortening	0.23	1.84	0.06	0.04	4.60	5.64	3.28	4.46	3.96	3.77	0.01	0.03	-	2.86	-
**Milk and dairy products**	13.55	11.86	15.19	17.76	12.25	22.55	7.57	2.64	10.72	12.56	11.02	23.97	0.27	13.48	-
Milks	9.50	5.72	11.13	9.44	4.95	9.01	3.01	0.85	5.16	7.27	5.94	13.86	-	6.53	-
Cheeses	0.67	2.25	0.46	4.00	4.04	7.17	2.42	1.02	2.95	2.13	0.20	0.47	-	3.28	-
Yogurt and fermented milk	2.92	3.07	3.16	3.77	2.40	4.72	1.62	0.55	2.35	2.86	3.86	7.71	0.26	2.18	-
Other dairy products	0.47	0.82	0.44	0.55	0.87	1.65	0.52	0.22	0.26	0.31	1.02	1.93	0.02	1.49	-
**Fish and shellfish**	4.02	4.57	2.93	14.32	5.14	4.12	3.23	10.24	12.34	31.36	0.05	0.05	-	17.13	-
**Meat and meat products**	6.18	12.83	4.85	28.81	19.44	23.12	18.44	17.93	35.34	33.65	0.16	0.30	-	31.47	-
Meat	4.47	8.30	3.54	19.24	12.01	14.38	11.49	11.00	21.94	24.58	0.00	0.01	-	21.92	-
Sausages and other meat products	1.55	4.35	1.15	9.02	7.25	8.49	6.81	6.84	13.04	8.82	0.16	0.29	-	8.59	-
Viscera and spoils	0.16	0.18	0.16	0.54	0.18	0.25	0.13	0.09	0.36	0.25	0.01	-	-	0.96	-
**Eggs**	1.74	2.69	1.53	5.75	4.70	5.05	3.65	5.26	2.89	2.04	0.00	0.02	-	24.81	-
**Pulses**	0.82	2.81	0.20	4.21	0.59	0.12	0.36	1.43	0.72	0.03	4.16	0.63	10.64	-	-
**Sugars and sweets**	0.64	2.64	0.13	0.27	0.44	0.81	0.32	0.15	0.08	0.07	5.85	12.97	0.62	0.10	-
Sugar	0.29	1.37	0.01	0.01	-	-	-	-	-	-	3.25	7.51	-	-	-
Chocolates	0.11	0.49	0.02	0.24	0.44	0.81	0.32	0.15	0.08	0.07	0.72	1.60	0.19	0.10	-
Jams and other	0.23	0.76	0.10	0.02	-	-	-	-	-	-	1.83	3.76	0.43	-	-
Other sweets	0.01	0.02	0.00	0.00	0.00	-	-	-	-	-	0.05	0.09	-	-	-
**Appetizers**	0.15	0.32	0.13	0.10	0.60	0.51	0.77	0.46	0.33	0.13	0.19	0.08	0.63	0.08	-
**Ready-to-eat meals**	3.09	1.79	3.57	1.96	2.00	2.34	1.71	2.21	2.13	2.20	1.74	0.73	1.41	0.74	-
**Sauces and condiments**	0.35	0.84	0.31	0.38	1.69	1.13	2.04	1.96	1.49	0.50	0.18	0.21	0.91	0.88	-
**Non-alcoholic beverage**	32.51	2.19	40.77	1.51	0.34	0.20	0.18	0.79	-	-	4.35	9.64	0.37	-	-
Water	22.11	-	27.78	-	-	-	-	-	-	-	-	-	-	-	-
Coffee and infusions	5.68	0.29	7.28	0.74	0.02	-	-	-	-	-	0.51	1.20	-	-	-
Sugar soft drinks	1.38	0.66	1.64	-	-	-	-	-	-	-	1.56	3.37	-	-	-
Non-sweetened soft drinks	0.72	0.01	0.95	0.00	0.02	-	-	-	-	-	0.01	0.02	-	-	-
Sports drinks	0.06	0.02	0.07	-	-	-	-	-	-	-	0.05	0.13	-	-	-
Energy drinks	-	-	-	-	-	-	-	-	-	-	-	-	-	-	-
Juices and nectars	1.58	0.91	1.83	0.24	0.01	-	-	-	-	-	1.99	4.38	0.28	-	-
Other drinks	0.99	0.30	1.23	0.53	0.30	0.20	0.18	0.79	-	-	0.24	0.54	0.08	-	-
**Alcoholic beverages**	5.17	3.54	6.62	0.28	-	-	-	-	-	-	1.15	3.40	-	-	100.00
Low alcohol content beverages	5.05	3.28	6.48	0.28	-	-	-	-	-	-	1.11	3.28	-	-	95.45
High alcohol content beverages	0.11	0.26	0.15	-	-	-	-	-	-	-	0.04	0.12	-	-	4.55
**Supplements and meal replacement**	0.02	0.09	-	0.29	0.02	0.03	-	-	-	-	0.03	0.01	0.11	0.06	-
